# Neural Threat Processing in Individuals with Small-Animal Phobia Is Shaped by Cluster C Personality Traits

**DOI:** 10.3390/ejihpe16090126

**Published:** 2026-08-26

**Authors:** Ascensión Fumero, Francisco Rivero, Rosario J. Marrero, Alessandro Grecucci, Teresa Olivares, Wenceslao Peñate

**Affiliations:** 1 Departamento de Psicología Clínica, Psicobiología y Metodología, Facultad de Psicología, Universidad de La Laguna, 38200 La Laguna, Tenerife, Spain; rmarrero@ull.edu.es (R.J.M.); tolivarp@ull.edu.es (T.O.); wpenate@ull.es (W.P.); 2 Instituto Universitario de Neurociencia (IUNE), Universidad de La Laguna, 38200 La Laguna, Tenerife, Spain; 3 Facultad de Ciencias de la Salud, Universidad Europea de Canarias, 38300 La Orotava, Tenerife, Spain; franciscoluis.rivero@universidadeuropea.es; 4 Department of Education, Psychology and Communication Sciences, University of Bari, 70122 Bari, Italy; alessandro.grecucci@uniba.it

**Keywords:** small-animal phobias, fMRI, threat processing, personality traits, Cluster C personality features

## Abstract

Small-animal phobia (SAP) is characterized by exaggerated fear responses to biologically relevant threat cues, yet the extent to which personality traits influences neural threat processing remains unclear. This study examined whether Cluster C personality traits (avoidant, dependent, and obsessive–compulsive) modulate neural responses to phobic stimuli in SAP. Functional magnetic resonance imaging (fMRI) data were collected from 39 individuals with SAP and non-phobic controls while viewing phobia-relevant and neutral stimuli. Whole-brain analyses were conducted to identify group differences in brain activation, and additional models assessed the influence of Cluster C personality traits on neural responses. Compared with controls, individuals with SAP showed greater activation in regions involved in threat processing and emotional salience, including the anterior cingulate cortex, parahippocampal gyrus, pallidum, fusiform gyrus, and thalamus. Higher avoidant traits were associated with increased activation in visual regions implicated in threat detection, whereas dependent and obsessive–compulsive traits were linked to greater recruitment of prefrontal areas involved in cognitive control and emotion regulation. These findings suggest that neural responses to phobic stimuli are influenced not only by the presence of SAP but also by personality-related vulnerability, highlighting the role of Cluster C traits in shaping perceptual and regulatory aspects of threat processing.

## 1. Introduction

Specific phobias are among the most prevalent anxiety disorders and are characterized by intense and disproportionate fear responses to circumscribed stimuli that pose little or no objective threat (American Psychiatric Association, 2022). Despite their apparent simplicity, specific phobias represent a powerful model for studying the neural mechanisms of threat processing because they involve rapid and highly selective fear responses to biologically salient cues. Importantly, these conditions are associated with marked functional impairment, as avoidance behaviors often generalize across contexts and can substantially interfere with daily functioning and social participation. Understanding the neural mechanisms underlying these responses is therefore essential for clarifying how phobic stimuli are detected, evaluated, and regulated by the brain. Converging evidence from neuroimaging research indicates that phobic responses arise from alterations in distributed brain circuits involved in threat detection, emotional salience, and regulatory control. In particular, limbic and paralimbic regions such as the amygdala, insula, thalamus, and parahippocampal cortex are consistently implicated in the rapid detection and evaluation of threatening stimuli. At the same time, prefrontal regions, including the medial and dorsolateral prefrontal cortex, play a critical role in top–down modulation of these threat responses by supporting processes such as cognitive reappraisal and fear inhibition (Wager et al., 2008; Duval et al., 2015). Dysfunction within this limbic–prefrontal circuitry has been repeatedly observed in individuals with specific phobias, who typically show exaggerated activation in threat-sensitive regions together with alterations in regulatory prefrontal systems (Ipser et al., 2013; Linares et al., 2012). These findings suggest that phobic reactions reflect not only heightened threat detection but also differences in the neural mechanisms that regulate emotional responses. Small-animal phobia (SAP) represents one of the most common forms of specific phobia. Individuals with SAP experience intense fear responses when confronted with small animals such as spiders, cockroaches, or lizards, often accompanied by strong avoidance behaviors and intrusive threat-related thoughts (LeBeau et al., 2010). Because these stimuli reliably trigger robust fear responses, SAP has been widely used as a model condition to investigate the neural architecture of fear processing. Neuroimaging studies of SAP have consistently reported heightened activity in regions involved in emotional salience and memory processing, including the amygdala, insula, thalamus, fusiform gyrus, and anterior cingulate cortex, supporting the view that phobic stimuli engage both perceptual and affective components of the brain’s threat detection system.

Research on the neurobiological mechanisms underlying small-animal phobia (SAP) has consistently implicated distributed cortical and subcortical circuits involved in threat detection, emotional salience, and regulatory control. Neuroimaging studies indicate that exposure to phobic stimuli robustly engages limbic structures such as the amygdala and insula, which play a central role in the rapid detection and evaluation of biologically relevant threats (Peñate et al., 2017). At the same time, cortical regions involved in higher-order cognitive and regulatory processes, including the dorsolateral prefrontal cortex, anterior cingulate cortex, and orbitofrontal cortex, have been shown to participate in the modulation and appraisal of these fear responses. Additional involvement of perceptual and associative regions such as the fusiform gyrus, occipital cortices, and inferior temporal areas further suggests that phobic stimuli recruit both affective and perceptual components of the brain’s threat processing system (Rivero et al., 2017). Beyond functional alterations, structural neuroimaging findings also support the presence of widespread neural differences in individuals with SAP. For instance, morphometric studies have identified structural variability in regions including the cerebellum, temporal pole, orbitofrontal cortex, amygdala, and thalamus—features that have recently been shown to reliably discriminate individuals with SAP from healthy controls using machine learning approaches (Scarano et al., 2024). Taken together, converging evidence from functional and structural neuroimaging suggests that SAP is associated with alterations across multiple neural systems involved in emotional processing, cognitive control, and perceptual threat evaluation (Rosenbaum et al., 2020).

However, an important question remains unresolved: whether the variability observed in these neural responses reflects differences in the core mechanisms of fear processing itself or, alternatively, individual differences in psychological vulnerability that modulate how threatening stimuli are perceived and regulated.

An important and still unresolved question concerns the extent to which neural responses to phobic stimuli are determined solely by the presence of a phobic disorder or are also shaped by stable individual differences in personality vulnerability. Although specific phobias are typically conceptualized as stimulus-driven fear responses, accumulating evidence suggests that trait-level psychological factors may influence how threatening stimuli are perceived, evaluated, and regulated at the neural level. In particular, personality configurations characterized by heightened anxiety sensitivity and avoidance may bias both perceptual vigilance to threat and the regulatory mechanisms that control emotional responses (Arena et al., 2025; Grecucci et al., 2025). Within this framework, Cluster C personality traits, comprising avoidant, dependent, and obsessive–compulsive personality features, represent a particularly relevant vulnerability domain. These personality configurations are characterized by heightened sensitivity to potential threat, fear of negative outcomes, and difficulties managing distressing emotional states. As a result, individuals with Cluster C traits often show persistent patterns of avoidance, hypervigilance, and maladaptive coping strategies when confronted with perceived threats (Friborg et al., 2013; Johnson & Vanwoerden, 2021; Arena et al., 2025; Grecucci et al., 2025). More broadly, these traits are associated with enduring emotional and interpersonal difficulties that frequently co-occur with anxiety disorders and may exacerbate the intensity and persistence of fear responses (Hofmann et al., 1995; Zarnowski et al., 2021). Similar vulnerability effects of personality pathology have been reported in other forms of psychopathology, including post-traumatic stress disorder, where personality disorder traits contribute significantly to symptom severity and maintenance (António et al., 2025). Likewise, evidence from emerging clinical domains suggests that personality disorder traits contribute to vulnerability across a broad range of psychological conditions, including problematic internet use and related behavioral disturbances, reinforcing their transdiagnostic relevance (Duradoni et al., 2025).

Consistent with this view, research has shown that anxious personality traits are associated with increased vulnerability to fear conditioning and fear generalization (Sep et al., 2019), two mechanisms that play a central role in the development and maintenance of phobic disorders. Neuroimaging studies further indicate that personality pathology is associated with alterations in limbic and paralimbic circuits involved in emotional salience and regulatory control (Zarnowski et al., 2021). Despite these conceptual links, however, the potential role of Cluster C personality traits in modulating neural responses to phobic stimuli has received little direct empirical investigation. Among Cluster C personality configurations, avoidant personality disorder (APD) may be particularly relevant for understanding individual differences in neural threat processing. APD is characterized by pervasive feelings of inadequacy, hypersensitivity to negative evaluation, and strong tendencies toward avoidance of potentially threatening situations (American Psychiatric Association, 2022). Neuroimaging research suggests that individuals with avoidant personality features exhibit heightened emotional reactivity to threatening stimuli, including increased amygdala responsiveness, while activity in prefrontal regulatory regions may remain relatively preserved (Denny et al., 2015). This pattern suggests a neural profile marked by enhanced threat sensitivity rather than deficits in regulatory capacity. Consistent with this interpretation, avoidant traits have been linked to social phobia (Pellecchia et al., 2018), and genetic studies indicate shared vulnerability factors between avoidant personality disorder and social anxiety (Reichborn-Kjennerud et al., 2007). However, despite these links with socially relevant threat processing, the potential contribution of avoidant personality features to neural responses to non-social phobic stimuli, such as small animals, remains largely unexplored. Obsessive–compulsive personality disorder (OCPD) represents another Cluster C configuration that may influence neural responses to threat-related stimuli. OCPD is characterized by rigid internal standards, perfectionism, and a strong need for cognitive and interpersonal control (American Psychiatric Association, 2022). Neuroimaging studies have reported alterations in spontaneous brain activity within regions involved in self-referential processing, cognitive control, and emotional regulation, including the precuneus, insula, caudate, and medial superior frontal cortex (Lei et al., 2020). At the same time, reduced activity in visual association regions such as the lingual and fusiform gyri has been interpreted as reflecting an excessive focus on external details. These findings suggest that individuals with obsessive–compulsive personality traits may recruit regulatory and control-related neural systems differently when confronted with emotionally salient stimuli, potentially shaping the neural processing of phobic cues. Dependent personality disorder (DPD) may also contribute to variability in emotional responses to threat. DPD is characterized by a pervasive need for reassurance and support from others, fear of abandonment, and difficulties making autonomous decisions (American Psychiatric Association, 2022). Although the neural basis of dependent personality traits remains relatively understudied, emerging neuroimaging findings suggest structural alterations in regions implicated in sensory and affective processing, including the postcentral gyrus and cuneus (Cui et al., 2024). More broadly, relatively few functional neuroimaging studies have examined neural activity patterns in individuals with dependent personality traits (Zarnowski et al., 2021). Consequently, the potential influence of dependent vulnerability on neural responses to phobic stimuli remains largely unknown. Taken together, these findings suggest that Cluster C personality traits may influence both perceptual and regulatory components of threat processing. Yet, despite strong theoretical links between anxious personality traits and fear-related neural systems, it remains unclear whether variability in avoidant, dependent, and obsessive–compulsive personality traits modulate the neural circuitry engaged during exposure to phobia-relevant stimuli. The primary objective of the present study was to examine differences in brain activation between individuals with small-animal phobia (SAP) and non-phobic healthy controls during the processing of phobic versus neutral stimuli. The secondary objective was to determine whether Cluster C personality traits modulate the neural responses associated with phobic stimulus processing. Using functional magnetic resonance imaging (fMRI), we investigated whether individual differences in avoidant, dependent, and obsessive–compulsive personality features were associated with differences in neural responses to phobic stimuli. Based on prior research on the neural circuitry of fear and personality-related vulnerability, we formulated four main hypotheses. First, consistent with previous neuroimaging studies of specific phobias, we expected individuals with SAP to show enhanced activation in neural circuits involved in threat detection and emotional salience, including the anterior cingulate cortex, parahippocampal regions, pallidum, and thalamus. Second, we hypothesized that higher levels of avoidant personality traits would be associated with increased activation in neural systems involved in threat detection and perceptual vigilance, reflecting heightened sensitivity to phobic cues. Third, we predicted that dependent personality traits would be associated with altered activation in regions involved in bodily and sensory aspects of emotional processing, including areas related to interoceptive awareness such as the postcentral gyrus and cuneus (Cui et al., 2024). Finally, we hypothesized that obsessive–compulsive personality traits would be associated with greater engagement of prefrontal regulatory systems involved in cognitive control and emotion regulation, reflecting attempts to modulate the emotional impact of phobic stimuli (Lei et al., 2020). Together, these hypotheses test the broader idea that neural responses to phobic stimuli are not determined solely by the presence of a phobia but are also shaped by trait-level personality vulnerability that modulates both perceptual and regulatory components of the brain’s threat processing system.

## 2. Materials and Methods

### 2.1. Participants

Thirty-nine adults (23 female) aged between 18 and 56 years (M = 29.41, SD = 11.82) participated in the study. Of these, 18 individuals met diagnostic criteria for small-animal phobia (SAP), while 21 participants served as non-phobic healthy controls (see Table 1 for descriptive statistics). Despite the relatively modest sample size, the study was adequately powered to detect the main effects of interest. A sensitivity analysis performed in G*Power 3.1. for a two-tailed independent-samples design (α = 0.05, power = 0.80) showed that, with 18 participants in the SAP group and 21 in the control group, the study was sensitive to effects of approximately d = 0.90 or larger. Given the use of clinically well-characterized groups and a hypothesis-driven design, the sample size was considered appropriate for detecting robust between-group effects. All participants provided written informed consent prior to participation, in accordance with institutional ethical guidelines. Participants in the SAP group were recruited between 2016 and 2018 through advertisements in newspapers, online platforms, flyers, and local media outlets inviting individuals experiencing intense fear of small animals to participate in a neuroimaging study on phobia. The diagnosis of specific phobia was established through a multi-step assessment procedure combining standardized questionnaires assessing phobia-related symptoms and anxiety levels with a semi-structured clinical interview conducted by trained researchers. To ensure diagnostic specificity, SAP had to represent the primary psychological condition, and participants were excluded if they presented with other current psychiatric or neurological disorders. The control group consisted of non-phobic individuals recruited primarily from the university community. These participants reported no history of specific phobias and showed low scores on the SAP assessment questionnaire. Control participants were matched to the SAP group in terms of gender distribution, although the SAP group was on average older than controls. Age was therefore included as a covariate in subsequent neuroimaging analyses to account for potential age-related variability in neural responses. Additional inclusion criteria for all participants included the absence of contraindications for magnetic resonance imaging (MRI), such as non-removable metal implants or other MRI-incompatible medical conditions. Participants were also required to be right-handed and to have normal or corrected-to-normal vision. Importantly, individuals in the SAP group were not undergoing psychological treatment for their phobia at the time of the study in order to avoid potential confounding effects of ongoing therapeutic interventions. Following participation in the study, participants with phobia were offered an eight-session psychological treatment program targeting their specific phobia. Participants who did not meet the inclusion criteria during the screening procedure were excluded prior to scanning.

### 2.2. Stimuli

To elicit robust and ecologically valid fear responses, participants were presented with dynamic visual stimuli depicting small animals commonly associated with specific phobias. The experimental paradigm consisted of a block design including 16 blocks of phobia-relevant stimuli and 16 blocks of neutral stimuli. Each block lasted 20 s. Phobic stimuli consisted of high-resolution video clips showing small animals in motion, including spiders, cockroaches, and lizards. These species were selected because they represent some of the most frequently reported stimuli in small-animal phobia and are known to reliably evoke fear responses in affected individuals. Importantly, phobic stimuli were individually tailored so that each participant viewed videos corresponding to the specific animal category associated with their own phobia. This individualized stimulus selection was implemented to maximize the emotional salience and ecological validity of the experimental manipulation. Neutral stimuli consisted of videos depicting wooden balls presented against a white background. These stimuli were selected to provide visually simple, emotionally neutral content while maintaining comparable visual characteristics across conditions. Both phobic and neutral stimuli were presented against an identical white background in order to minimize potential confounds related to low-level visual features such as color, luminance, or scene complexity. All stimuli were presented using stereoscopic 3D video through MRI-compatible VisualStim Digital goggles (Resonance Technology, Inc., Northridge, CA, USA), with a GeForce 8600GT graphics card. The use of dynamic stereoscopic stimuli was intended to increase perceptual realism and enhance the engagement of neural systems involved in threat detection and emotional processing. The animals used in the video stimuli were filmed specifically for the experiment under controlled conditions and were subsequently released back into their natural environment. Stimulus presentation followed a randomized block order to reduce potential order effects and anticipatory responses. Each participant viewed an equal number of phobic and neutral blocks, ensuring a balanced design across conditions.

### 2.3. Questionnaires

A set of standardized clinical and personality assessment instruments was administered in order to characterize participants with respect to phobia diagnosis, anxiety severity, and personality traits associated with Cluster C vulnerability. The Composite International Diagnostic Interview (CIDI, Version 2.1; Kessler & Üstün, 2004) was used to assess the presence of specific phobia and other anxiety-related conditions, including agoraphobia, social phobia, and panic attacks. The CIDI is a widely used structured diagnostic interview designed to assess psychiatric disorders according to standardized diagnostic criteria. In the present study, the instrument was used as part of the diagnostic procedure to confirm the presence of small-animal phobia and to exclude other primary anxiety disorders. Sociodemographic information, including age and sex, was also collected. To quantify the severity of anxiety symptoms, participants completed the Hamilton Anxiety Rating Scale (HARS; Hamilton, 1959; Bruss et al., 1994). The HARS is a clinician-administered 14-item scale that assesses psychological and somatic symptoms of anxiety using a 5-point Likert-type scale ranging from 0 (not present) to 4 (very severe). The scale has demonstrated good inter-rater reliability, with intraclass correlation coefficients ranging from 0.74 to 0.96 (Bruss et al., 1994; Thompson, 2015). In the present study, the HARS was used to characterize the severity of anxiety symptoms associated with phobic responses. Personality traits associated with Cluster C personality vulnerability were assessed using the International Personality Disorder Examination (IPDE; Loranger et al., 1997). The IPDE is a standardized instrument designed to evaluate personality disorder traits according to established diagnostic frameworks. In the present study, the 24 items corresponding to Cluster C personality features (avoidant, dependent, and obsessive–compulsive traits) were administered using a true/false response format. The IPDE has been widely used in both clinical and research contexts and shows adequate psychometric properties, with reported interrater reliability coefficients ranging from 0.84 to 0.92 (Lenzenweger et al., 2007; Esbec & Echeburúa, 2014). These measures were used to quantify individual variability in Cluster C personality traits for subsequent analyses examining their modulatory role in neural responses to phobic stimuli. Participants’ behavioral responses to phobia-related situations were assessed using the Situation–Response (S–R) Inventory of Anxiousness (Endler et al., 1962). This inventory consists of 14 items rated on a 5-point Likert scale and measures physiological, cognitive, and behavioral responses to anxiety-provoking situations, including exposure to phobia-relevant stimuli such as spiders, cockroaches, lizards, or mice. The S–R Inventory has demonstrated high internal consistency (α ≈ 0.95) and adequate convergent validity (Kameoka & Tanaka-Matsumi, 1981). In the present study, this instrument was administered to both groups to assess phobia-related anxiety responses. Finally, the Edinburgh Handedness Inventory (Oldfield, 1971) was administered to confirm right-handedness in all participants. This 10-item forced-choice questionnaire assesses hand preference across a range of everyday activities and is commonly used in neuroimaging research to control for potential variability associated with hemispheric lateralization.

### 2.4. Procedure

Participants who met the inclusion criteria were invited to the laboratory for the experimental session. Upon arrival, the study procedures were explained in detail, and written informed consent was obtained from all participants. Subsequently, participants completed the clinical and personality assessment instruments described above. Prior to scanning, participants received standardized instructions regarding the experimental task and were familiarized with the MRI environment in order to reduce potential anxiety and movement during the acquisition. Because MRI scanning requires careful positioning and calibration, an initial preparation phase lasting approximately 15 min was conducted to ensure proper head stabilization, optimal visual alignment with the display system, and correct functioning of the stimulus presentation equipment. During the fMRI session, participants completed a task-based paradigm in which phobia-related and neutral stimuli were presented in a block design. Functional brain activity was recorded through blood-oxygen-level-dependent (BOLD) signals while participants viewed the stimuli. The experimental paradigm consisted of alternating blocks of phobic and neutral stimuli. In total, participants were presented with 32 blocks, including 16 blocks of phobia-relevant stimuli and 16 blocks of neutral stimuli. Each block lasted 20 s. Phobia-related blocks consisted of videos depicting the specific animal category associated with each participant’s phobia (e.g., spiders, cockroaches, or lizards), whereas neutral blocks consisted of videos showing wooden balls. Stimuli were presented in a pseudo-randomized order to minimize potential order effects and anticipatory responses. All stimuli were presented against an identical white background in order to control for potential low-level visual differences across conditions. The entire functional acquisition, including stimulus presentation and image recording, lasted approximately 20 min. Throughout the scanning session, participants were instructed to remain as still as possible and to attentively watch the stimuli presented through the MRI-compatible visual display system. The study was conducted within a broader line of research in accordance with the ethical standards of the Declaration of Helsinki and was covered by successive ethical approvals granted by the Ethics Committee for Research and Animal Welfare of the University of La Laguna, Spain, from 2013 (CEIBA2013-0086) to 2017 (CEIBA2017-0245).

### 2.5. Design

The study employed a between-group experimental design comparing individuals with small-animal phobia (SAP) and non-phobic healthy controls. In addition to group differences, the experimental paradigm included a within-subject manipulation of stimulus type, allowing the comparison of neural responses to phobia-relevant versus neutral stimuli. All participants underwent a single task-based fMRI session during which they were presented with blocks of phobic and neutral stimuli. Phobic stimuli consisted of videos depicting small animals associated with the participants’ specific phobia (e.g., spiders, cockroaches, or lizards), whereas neutral stimuli consisted of visually comparable videos depicting wooden balls. This design enabled the examination of both between-group differences in threat processing and within-subject neural responses to emotionally salient versus neutral stimuli. The primary dependent variable was brain activation measured through changes in the blood-oxygen-level-dependent (BOLD) signal recorded during the presentation of the stimuli. The primary outcome was the between-group difference in BOLD responses to phobic versus neutral stimuli. Secondary outcomes concerned the association between BOLD responses to phobic versus neutral stimuli and each of the three Cluster C personality traits: avoidant, dependent, and obsessive–compulsive traits. Whole-brain analyses were conducted to identify neural regions showing differential activation as a function of stimulus type and group membership. In addition, individual differences in Cluster C personality traits were examined in relation to neural responses to phobic stimuli.

### 2.6. fMRI Data Acquisition

Whole-brain functional magnetic resonance imaging (fMRI) data were acquired using a 3.0 Tesla GE Sigma Excite HD scanner equipped with a 12-channel head coil. Participants were instructed to remain as still as possible during the acquisition and to attentively view the stimuli presented through the MRI-compatible visual display system. Functional images were collected using a T2-weighted gradient-echo echo-planar imaging (EPI) sequence* sensitive to blood-oxygen-level-dependent (BOLD) contrast. Acquisition parameters were as follows: repetition time (TR) = 2000 ms, echo time (TE) = 30 ms, flip angle (FA) = 75°, field of view (FOV) = 25.6 cm, matrix size = 64 × 64, and 32 axial slices covering the whole brain. The resulting voxel size was 4 × 4 × 4 mm^3^. High-resolution structural images were acquired for anatomical localization and spatial normalization using a T1-weighted three-dimensional fast spoiled gradient echo (FSPGR) sequence with the following parameters: TR = 8852 ms, TE = 1.756 ms, flip angle = 10°, field of view = 25.6 cm, matrix size = 256 × 256, and 172 sagittal slices, resulting in an isotropic voxel size of 1 × 1 × 1 mm^3^. Stimuli were presented using an MRI-compatible VisualStim digital display system (Resonance Technology, Inc., Northridge, CA, USA) connected to a computer equipped with an NVIDIA GeForce 8600 GT graphics card. Participants viewed the stimuli through MRI-compatible stereoscopic goggles, allowing the presentation of three-dimensional visual stimuli within the scanner environment. To ensure signal stabilization, the initial volumes of each functional run were discarded prior to analysis. This standard procedure removes the transient signal fluctuations typically observed at the beginning of echo-planar image acquisition and ensures that only steady-state BOLD signals are included in the statistical analyses. All structural and functional images were visually inspected by an experienced neuroradiologist prior to preprocessing in order to identify potential artifacts, excessive motion, or structural abnormalities.

### 2.7. fMRI Processing and Analyses

Functional imaging data were analyzed using SPM12 (revision 7219; Statistical Parametric Mapping; Wellcome Department of Imaging Neuroscience, London, UK) implemented in MATLAB R2017b (MathWorks, Natick, MA, USA). Imaging data were first converted from DICOM to NIfTI format and visually inspected for potential artifacts. Prior to preprocessing, both functional and structural images were manually reoriented to the anterior commissure–posterior commissure (AC–PC) plane to ensure consistent anatomical alignment across participants. Standard preprocessing procedures implemented in SPM12 were then applied. These steps included motion correction (realignment), co-registration of functional and structural images, segmentation of anatomical images, spatial normalization to the Montreal Neurological Institute (MNI) template, and spatial smoothing using an isotropic Gaussian kernel of 8 mm full width at half maximum (FWHM). At the first (subject) level, neural responses to the experimental conditions were modeled using the general linear model (GLM) implemented in SPM12. The experimental conditions (phobic stimuli and neutral stimuli) were modeled as block regressors and convolved with the canonical hemodynamic response function (HRF). Motion parameters obtained during realignment were included in the model as nuisance regressors to account for residual movement-related variance. Contrast images were generated for each participant to examine differences in neural activation between phobic and neutral conditions. At the second (group) level, contrast images from individual participants were entered into random-effects analyses to examine group-level effects. Age was entered as a continuous covariate at the second-level general linear models, with one age value specified for each participant. The default SPM12 centering option (“Overall mean”) was retained, resulting in mean-centering of age around the overall sample mean. Whole-brain analyses were conducted to compare neural responses between individuals with small-animal phobia (SAP) and non-phobic controls using the full sample. Statistical inference was based on a voxel-wise cluster-forming threshold of *p* < 0.001 (uncorrected) and a minimum cluster extent of k > 3 contiguous voxels. This relatively stringent primary threshold was selected for the exploratory whole-brain analyses, consistent with methodological recommendations for cluster-based fMRI analyses (Woo et al., 2014). The cluster extent criterion was applied as an additional requirement for spatial contiguity and was not intended to constitute an independent correction for multiple comparisons. Accordingly, the thresholding procedure does not provide family-wise error (FWE) or false discovery rate (FDR) control. Given the acquisition voxel size of 4 × 4 × 4 mm^3^, k > 3 corresponds to a minimum of four contiguous voxels, or approximately 256 mm^3^. The resulting whole-brain findings were therefore considered exploratory and interpreted cautiously. Anatomical localization of significant clusters was performed using the WFU PickAtlas toolbox (version 3.0.5; Maldjian et al., 2003) in combination with the Automated Anatomical Labeling atlas (AAL2; Rolls et al., 2015). To examine whether the pattern of neural differences between individuals with SAP and non-phobic controls varied as a function of Cluster C personality traits, three additional second-level whole-brain analyses were estimated. In each analysis, one Cluster C personality trait score (avoidant, dependent, or obsessive–compulsive) was entered as a continuous covariate. The same voxel-wise cluster-forming threshold of *p* < 0.001 (uncorrected) and minimum cluster extent of k > 3 contiguous voxels were applied. Separate models were estimated because the aim of the study was to identify the specific neural patterns observed in models including each Cluster C personality trait rather than to examine their combined or interactive effects. Consequently, the three personality traits were analysed independently. Interaction effects among Cluster C traits were not examined. Given the limited sample size of these within-group analyses, the resulting findings were considered exploratory and hypothesis-generating and were interpreted cautiously.

## 3. Results

### 3.1. Group Differences

Descriptive statistics for the main behavioral and clinical measures are reported in Table 1. As expected, individuals with small-animal phobia (SAP) showed significantly higher levels of anxiety-related responses compared to non-phobic controls. Specifically, the SAP group reported markedly higher scores on the Situation–Response Inventory of Anxiousness (S–R) (t(37) = 22.688, *p* < 0.001) as well as higher levels of general anxiety measured by the Hamilton Anxiety Rating Scale (HARS) (t(37) = 7.370, *p* < 0.001). These findings confirm the clinical validity of the group classification and indicate that the SAP participants exhibited substantially stronger behavioral and emotional responses to phobia-related stimuli. Importantly, no significant differences were observed between groups in Cluster C personality trait scores, including avoidant (t(37) = −0.197, *p* = 0.845), dependent (t(37) = −0.047, *p* = 0.963), and obsessive–compulsive traits (t(37) = 0.691, *p* = 0.494). Thus, the two groups did not differ significantly in Cluster C personality trait scores.

### 3.2. SAP Versus Controls

**Figure 1:**
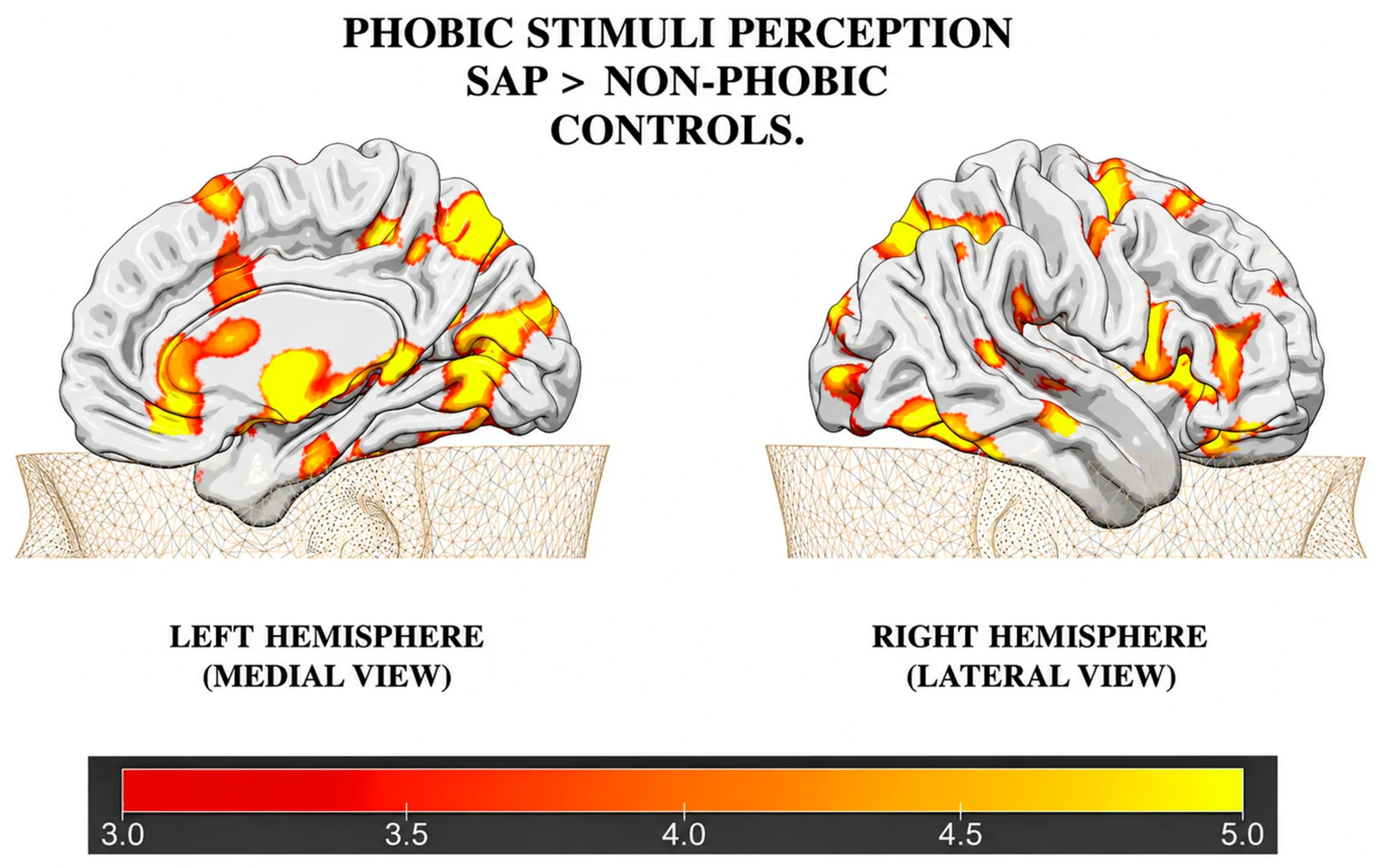
Neural responses to phobic stimuli in individuals with small-animal phobia (SAP) compared with non-phobic controls. The figure shows brain regions exhibiting greater activation in individuals with small-animal phobia compared with non-phobic controls during the perception of phobic stimuli. Increased BOLD responses were observed in regions associated with fear processing and emotional salience, including the anterior cingulate cortex, parahippocampal gyrus, pallidum, fusiform gyrus, and thalamus, among others (see Table 2 for full list of regions). The opposite contrast did not yield any significant activation.

Whole-brain activation results are summarized in Table 2. See also Figure 1 for depiction of brain activity. Individuals with SAP showed significantly greater neural responses to phobia-related stimuli compared with controls. These differences were observed across a distributed set of brain regions known to be involved in threat detection, emotional processing, and memory-related aspects of fear responses. In particular, participants with SAP exhibited bilateral activation in the anterior cingulate cortex, parahippocampal gyrus, pallidum, fusiform gyrus, and thalamus. Additional activations were observed in the left putamen, caudate nucleus, cerebellum and vermis, middle occipital cortex, middle temporal gyrus, and the pars triangularis of the inferior frontal gyrus.

### 3.3. Neural Responses to Phobic Stimuli as a Function of Cluster C Personality Traits

**Figure 2:**
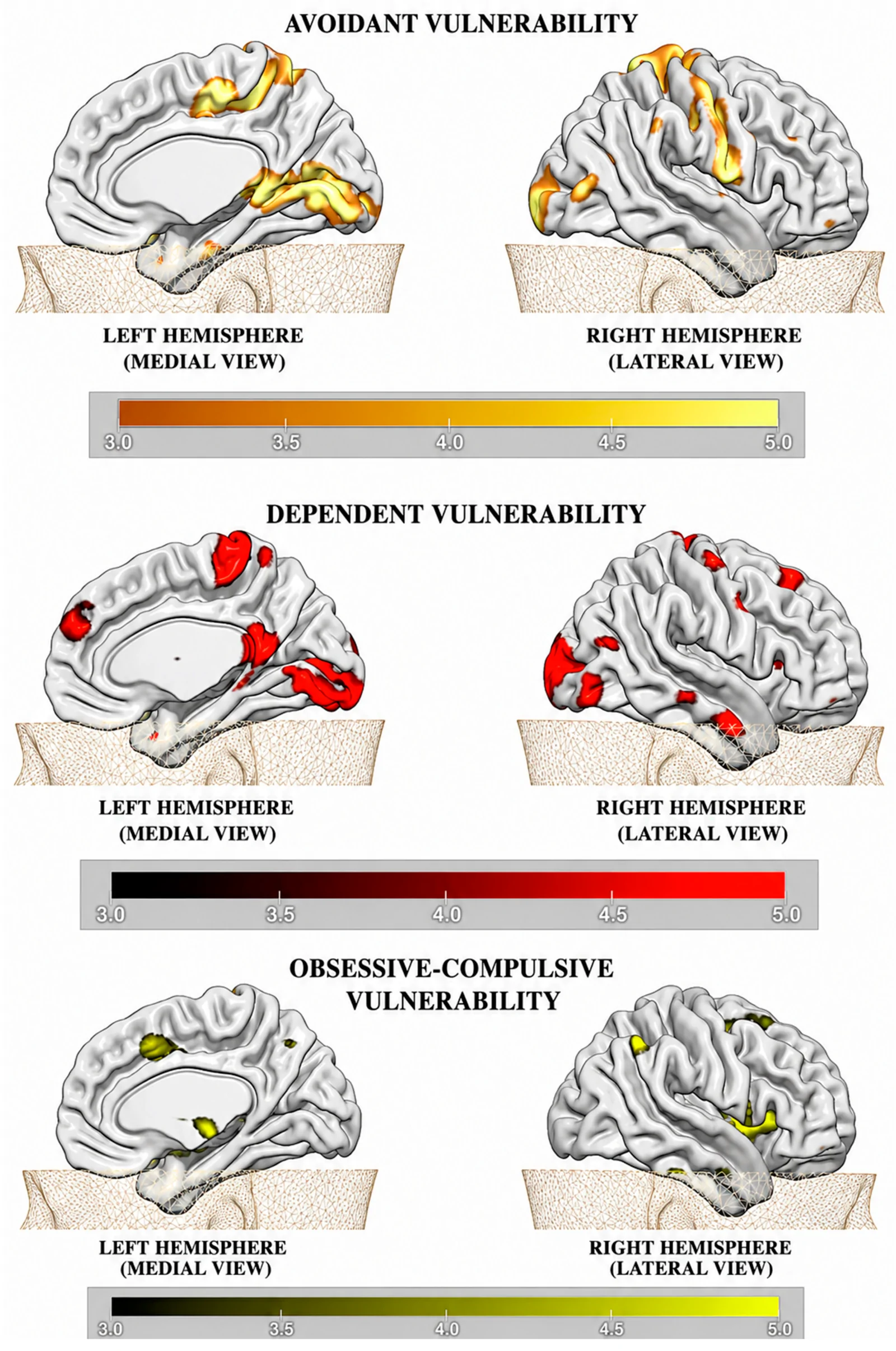
Whole-brain activation associated with Cluster C personality traits during phobic stimulus processing. Brain regions showing greater activation in second-level whole-brain models including continuous Cluster C personality trait scores as covariates for the contrast phobic stimuli > neutral stimuli and continuous scores for Avoidant Personality Disorder traits, Dependent Personality Disorder traits, and Obsessive–Compulsive Personality Disorder traits. Each panel represents a separate second-level whole-brain analysis in which one Cluster C personality trait score was entered as an independent continuous covariate. Separate statistical models were estimated for each personality dimension. Warmer colors indicate greater activation in models including personality trait scores. Coordinates and anatomical labels of significant clusters are reported in Table 3. Statistical maps are displayed at *p* < 0.001 (uncorrected), minimum cluster extent = 3 voxels.

Table 3 details the distinct patterns of neural activation identified for avoidant, dependent, and obsessive–compulsive personality traits during processing of phobic stimuli. Figure 2 illustrates the spatial distribution of the significant activations summarized in Table 3.

#### 3.3.1. Avoidant Personality Traits

When avoidant personality traits were included as a covariate, the pattern of neural activation associated with phobic stimuli shifted toward regions primarily involved in visual and perceptual processing of threat-related information. Compared with non-phobic controls, individuals with SAP showed greater activation in the bilateral lingual gyrus, calcarine cortex, and postcentral gyrus, as well as in the left hippocampus, right superior occipital cortex, and right fusiform gyrus. Additional activation was observed in the supplementary motor area and Rolandic operculum. Notably, fusiform gyrus activation remained consistently higher in individuals with phobia compared to controls, both in analyses including and excluding the avoidant personality covariate (see Figure 2), highlighting the robustness of visual threat-processing mechanisms in SAP.

#### 3.3.2. Dependent Personality Traits

When dependent personality traits were included as a covariate, the pattern of neural activation expanded to include regions associated with higher-order cognitive processing and emotional regulation. Significant activations were observed in the bilateral superior frontal cortex, middle frontal gyrus, precuneus, and insula, as well as in occipital regions including the calcarine cortex, lingual gyrus, and middle occipital gyrus. Additional activation was detected in the cerebellum, vermis, and paracentral lobule (see Figure 2).

#### 3.3.3. Obsessive–Compulsive Personality Traits

Finally, when obsessive–compulsive personality traits were included in the model, significant activations were observed in a network involving subcortical and frontal regions associated with cognitive control and action regulation. Compared with controls, individuals with SAP showed increased activation in the bilateral pallidum, bilateral thalamus, left putamen, left Rolandic operculum, and right cerebellum and vermis. Increased activation was also detected in the inferior frontal operculum (See Figure 2).

## 4. Discussion

The main aim of the present study was to characterize the neural responses to phobic stimuli in individuals with small-animal phobia (SAP) compared to healthy controls, and to examine whether Cluster C personality traits modulate these neural responses. The marked differences in phobia-related and general anxiety measures confirm the expected clinical distinction between participants with SAP and non-phobic controls. At the same time, the absence of significant group differences in avoidant, dependent, and obsessive–compulsive personality trait scores is relevant to the interpretation of the subsequent neuroimaging findings. Because the groups were comparable in Cluster C personality traits at the behavioral level, the neural differences observed in models including these traits can be considered in the context of individual variability in personality characteristics rather than differences in the mean levels of these traits between groups. This provides an important context for interpreting the personality-related neuroimaging analyses as examining individual differences in neural responses within the sample.

Our findings revealed that individuals with SAP showed heightened activation in several regions involved in visual, emotional, and thereat-related processing, including the anterior cingulate cortex, parahippocampal region, pallidum, fusiform gyrus, and thalamus. These results are consistent with previous neuroimaging studies of specific phobias reporting increased activity in cortico–limbic and subcortical circuits involved in threat detection, emotional appraisal, and the preparation of defensive responses. Beyond these group differences, our findings provide novel evidence that Cluster C personality traits are related to differences in neural response to phobic stimuli. When statistically controlling for avoidant, dependent, and obsessive–compulsive personality tendencies, the pattern of brain activation associated with phobic stimuli changed in systematic ways, indicating that trait-level personality characteristics are related to differences in the neural systems recruited during the processing of phobic stimuli. Importantly, these findings suggest that phobic responses cannot be understood solely as stimulus-driven reactions within a fixed fear circuitry. Instead, they appear to emerge from the interaction between stimulus-related threat processing and stable personality dispositions that influence how threat-related information is perceived, evaluated, and regulated. In this sense, anxious personality traits may amplify or reshape neural responses within fear-related networks, making threat-processing systems more reactive in individuals with higher vulnerability. Taken together, the present results contribute to a growing body of literature indicating that the neural mechanisms underlying phobic reactions are not only determined by the characteristics of the feared stimulus, but are also shaped by trait-level personality configurations that bias the processing of threat signals.

In the following sections, we discuss how specific Cluster C traits, avoidant, dependent, and obsessive–compulsive, may differentially shape perceptual, emotional, and regulatory components of the threat response.

### 4.1. The Role of Cluster C Personality Traits in Threat Perception

The present findings suggest that Cluster C personality traits are associated with distinct components of the neural systems involved in threat processing. Rather than reflecting a unitary neural response, the processing of phobic stimuli appears to involve multiple functional systems—including perceptual threat detection, emotional evaluation, and regulatory control—whose engagement may vary depending on individual personality traits. These findings suggest that the involvement of visual perceptual processing in SAP is not solely attributable to individual differences in avoidant personality traits and may represent a more general feature of the neural response to phobic stimuli.

The pattern associated with avoidant personality traits was predominantly characterized by activation of regions involved in visual and perceptual processing, whereas dependent personality traits were associated with a more distributed pattern involving regions implicated in higher-order cognitive and emotional processing. In contrast, obsessive–compulsive personality traits were associated with activation of subcortical and frontal regions involved in behavioral and regulatory control. The following sections consider these patterns separately and discuss their potential implications for the neural processing of phobic stimuli.

#### 4.1.1. Avoidant Traits and Perceptual Threat Vigilance

When examining the association between avoidant personality traits and neural responses to phobic stimuli, increased activation was observed in several regions involved in visual and perceptual processing, including the bilateral lingual gyrus, calcarine cortex, postcentral gyrus, left hippocampus, right superior occipital cortex, and right fusiform gyrus. Additional activation was observed in the supplementary motor area and Rolandic operculum. Taken together, this pattern suggests that avoidant personality traits may be particularly relevant to the perceptual processing of phobia-related cues. Several of these regions have been implicated in visual attention, visuospatial processing, sensory processing, and the identification of salient stimuli. The increased activation observed in these areas may therefore reflect heightened perceptual vigilance toward phobia-relevant cues in individuals with stronger avoidant personality traits.

This interpretation is consistent with theoretical models proposing that avoidance-related traits are associated with enhanced attentional sensitivity to potential threats. The processing of threat signals may therefore rapidly activate action-related systems, promoting avoidance tendencies and defensive behaviors (Åhs et al., 2015). From this perspective, avoidant personality traits may amplify early perceptual stages of processing phobia-relevant cues, making them more salient and more rapidly detected. Previous research supports this interpretation. Studies have reported abnormal early activity in subcortical networks—including the amygdala, hippocampus, and insula—in individuals with avoidance personality traits, reflecting hypervigilance toward potential threats (Hofmann et al., 2012). Consistent findings also indicate increased amygdala reactivity in individuals with avoidant personality characteristics, highlighting their heightened sensitivity to threatening stimuli. Interestingly, despite this enhanced limbic reactivity, prefrontal activity appears to remain comparable to that of healthy individuals, suggesting that regulatory mechanisms may remain relatively preserved in these individuals (Denny et al., 2015). At the same time, previous work suggests that cognitive avoidance strategies may sometimes reduce neural responsiveness to threat. For instance, individuals with higher dispositional cognitive avoidance show diminished neural responses to threat-related distractors across several cortical and subcortical regions (Günther et al., 2023). This pattern suggests that while avoidant traits may increase perceptual vigilance to threat cues, certain forms of cognitive avoidance might also function as a protective strategy by limiting the depth of emotional processing of distressing stimuli.

Interestingly, fusiform gyrus activation remained higher in individuals with phobia in both analyses including and excluding the avoidant personality covariate. This finding suggests that the involvement of visual threat-processing mechanisms in SAP is not solely attributable to individual differences in avoidant personality traits and may represent a more general feature of the neural response to phobic stimuli.

#### 4.1.2. Dependent Traits and Emotional–Self-Referential Processing

A different pattern emerged in the analysis of dependent personality traits. Increased activation was observed in a distributed set of regions involving frontal, parietal, insular, occipital, and cerebellar areas, including the bilateral superior frontal cortex, middle frontal gyrus, precuneus, insula, calcarine cortex, lingual gyrus, middle occipital gyrus, cerebellum, vermis, and paracentral lobule. Additional involvement of the supramarginal gyrus, angular gyrus, and inferior frontal pars triangularis was also observed. Rather than being restricted to early perceptual processing, this distributed pattern suggests that dependent personality vulnerability may be associated with the recruitment of neural systems involved in the integration of perceptual, cognitive, and emotional information during the processing of phobic stimuli. Several of the regions involved, particularly the frontal and insular areas, have been implicated in higher-order cognitive processing and emotional regulation, whereas the occipital and parietal regions may contribute to the integration and evaluation of sensory information. Their joint involvement may therefore indicate that dependent personality traits influence how emotional meaning is assigned to threatening stimuli and how these experiences are cognitively evaluated and regulated. In particular, increased activation of the precuneus is of interest because this region has been consistently associated with self-referential processing and self-awareness. The present finding may therefore indicate that phobia-related stimuli engage self-focused processing to a greater extent in individuals with higher levels of dependent personality traits. However, this interpretation should be considered cautiously, as the present study did not directly assess self-referential processing during the fMRI task. The possible involvement of self-referential processing is consistent with previous work suggesting that self-focused cognitive processes may be particularly relevant in personality pathology and may contribute to emotional distress in threatening situations (Farmer & Chapman, 2012). Previous research has also reported alterations in decision-making and prefrontal regions involved in cognitive control in individuals with dependent personality disorder, including the inferior frontal gyrus (Tariq et al., 2023). Consistent with these observations, the present analysis showed increased activation in the inferior frontal pars triangularis. Although these findings do not establish a specific functional role for this region in dependent personality vulnerability, they are compatible with the involvement of prefrontal regulatory processes in the neural response to phobic stimuli.

Taken together, the pattern associated with dependent personality traits suggests that these characteristics may influence intermediate aspects of threat processing involving emotional evaluation, self-referential interpretation, and cognitive regulation. In this respect, the dependent pattern differs from the predominantly perceptual pattern observed in association with avoidant traits, suggesting that different Cluster C dimensions may be associated with partially distinct aspects of the neural response to phobic stimuli.

#### 4.1.3. Obsessive–Compulsive Traits and Regulatory Control

The neural patterns associated with obsessive–compulsive personality traits differed from those observed for avoidant and dependent traits. Increased activation was observed in the bilateral pallidum, bilateral thalamus, left putamen, left Rolandic operculum, right inferior frontal operculum, right cerebellum, and vermis. The involvement of these regions suggests that obsessive–compulsive personality traits may be associated with the recruitment of neural systems involved in the regulation and coordination of responses to phobia-related stimuli. The pallidum, putamen, and thalamus are core components of cortico–striatal–thalamo–cortical circuits involved in action selection and behavioral control, while frontal opercular and cerebellar regions contribute to the integration and regulation of cognitive, emotional, and motor processes. The engagement of these systems may therefore indicate that obsessive–compulsive vulnerability is particularly relevant to later stages of threat processing involving response regulation and inhibitory control. Within this framework, individuals with phobias who exhibit higher levels of obsessive–compulsive traits may experience greater difficulty regulating intrusive threat-related representations or suppressing maladaptive behavioral tendencies, potentially contributing to more pronounced phobic responses. This interpretation is consistent with evidence linking obsessive–compulsive personality traits to executive dysfunctions, particularly in working memory, attentional set-shifting, and planning (García-Villamisar & Dattilo, 2015).

However, the literature also highlights the heterogeneity of obsessive–compulsive traits. Nonclinical individuals with obsessive–compulsive personality disorder (OCPD) may show cognitive inflexibility and deficits in executive planning while maintaining relatively preserved decision-making abilities. Moreover, community samples with obsessive–compulsive personality traits, but without a clinical diagnosis of OCPD, may not show clear impairments in motor inhibition (Fineberg et al., 2015). These findings suggest that the relationship between obsessive–compulsive traits and cognitive control may vary according to trait severity and clinical expression.

The present findings should therefore be interpreted as evidence of an association between obsessive–compulsive personality traits and the recruitment of neural systems involved in regulatory and response-control processes, rather than as direct evidence of impaired inhibitory control. This distinction is particularly relevant because inhibitory control and related executive processes were not directly assessed during the present fMRI task.

Recent neuroimaging research in small-animal phobia has highlighted the involvement of large-scale neural systems supporting emotional regulation, cognitive control, and the integration of sensory and affective information. In particular, Arena et al. (2025) reported that networks including the anterior cingulate cortex, insula, and precuneus contributed to distinguishing individuals with small-animal phobia from healthy controls, emphasizing that phobic responses involve not only threat-detection mechanisms but also higher-order regulatory systems. Within this broader neurobiological framework, the present findings suggest that personality traits may influence different stages of neural threat processing. Avoidant traits appear to be primarily associated with perceptual vigilance and early threat processing, dependent traits with evaluative and self-referential aspects of threat processing, and obsessive–compulsive traits with regulatory and response-control processes. Rather than indicating completely separate mechanisms, these patterns may reflect partially distinct contributions of personality characteristics to the processing of fear-relevant stimuli across distributed neural networks.

More broadly, the present findings may also be considered within an emerging brain–body perspective on psychopathology. Emotional brain systems do not operate independently of systemic physiological processes, and recent multimodal evidence has identified coordinated relationships between brain alterations, circulating proteins, and metabolic dysfunction in depression (Lian et al., 2026). Although the present study did not assess metabolic, inflammatory, endocrine, or other peripheral physiological markers, the observed involvement of neural systems related to threat processing and emotional regulation may be considered within a broader framework in which persistent or recurrent phobic anxiety and associated personality traits interact with systemic physiological processes. From this perspective, repeated or sustained engagement of neural systems involved in threat detection and emotional regulation could represent one component of a more complex interaction between psychological distress and systemic physiology. This possibility extends the clinical relevance of the present findings beyond the characterization of cerebral responses to phobic stimuli while emphasizing the need for future studies combining neuroimaging with peripheral physiological and metabolic measures in specific phobia.

Beyond their neural patterns, these findings may have implications for understanding individual differences in the subjective experience of phobic situations. Although all participants with SAP were characterized by marked fear responses, Cluster C personality traits may contribute to differences in how phobic stimuli are perceived, experienced, and regulated. However, this interpretation remains speculative, as the present study did not directly assess participants’ subjective emotional experience during fMRI acquisition. Future studies combining neuroimaging with online subjective ratings or ecological assessments of fear and emotional responses could help clarify how personality-related neural differences translate into the lived experience of individuals with specific phobias.

Future research could build on the present findings by addressing both their reproducibility and their underlying biological mechanisms. Internal cross-validation procedures and independent samples could be used to assess the reproducibility, robustness, and generalizability of the neural patterns identified in the present study. In parallel, advances in precision genome engineering may provide new opportunities to investigate the molecular mechanisms contributing to individual differences in neural threat processing and emotional regulation (Huang et al., 2024). When integrated with human genetic, neuroimaging, and systems neuroscience approaches, these emerging methodologies could help bridge molecular mechanisms and systems-level neural phenotypes, providing a more comprehensive understanding of the biological pathways underlying vulnerability to specific phobias and Cluster C personality characteristics.

### 4.2. Limitations

Despite the strengths of the present study, several limitations should be acknowledged. First, the study examined Cluster C personality traits as continuous traits rather than recruiting participants with formally diagnosed comorbid personality disorders. Therefore, the present findings cannot be generalized to individuals presenting with both small-animal phobia and a full comorbid Cluster C personality disorder. The present dimensional approach was useful for examining individual differences in personality characteristics, but future studies should include clinically diagnosed comorbid samples to determine whether similar neural patterns are observed when personality pathology reaches a diagnostic threshold. Second, the substantial age difference between the SAP and control groups represents an additional limitation. Although age was included as a continuous covariate in the second-level analyses to statistically account for age-related variance in BOLD responses, covariate adjustment may not fully exclude residual or nonlinear age-related confounding when groups differ markedly in age. An age-matched sensitivity analysis could have further addressed this issue; however, such an approach would require reducing the available sample and consequently further limit statistical power. Therefore, the between-group findings should be interpreted as statistically adjusted for age rather than as entirely independent of age-related factors. Future studies with larger samples and closer age matching between clinical and control groups will be important to determine whether the observed neural differences are robust to demographic differences in age. Third, the study did not examine the potential contribution of individual differences in general anxiety severity to the observed neural responses. Although the Situation–Response Inventory of Anxiousness and the Hamilton Anxiety Rating Scale were used to characterize anxiety severity and establish the clinical distinction between the SAP and control groups, these measures were not included as continuous predictors or covariates in the neuroimaging models. Consequently, the between-group neural findings cannot be interpreted as entirely independent of differences in general anxiety severity, nor could dimensional associations between anxiety severity and BOLD responses be examined. Given the relatively small sample size, incorporating additional clinical predictors would have increased model complexity and reduced statistical power to detect the effects of interest, potentially increasing the risk of Type II errors. Future studies with larger samples could examine whether neural differences associated with SAP remain after accounting for individual differences in general anxiety severity and could directly test dimensional relationships between anxiety severity and neural responses to phobic stimuli. Fourth, the statistical thresholding strategy and sample size limit the robustness and generalizability of the whole-brain findings. Although a relatively stringent voxel-wise cluster-forming threshold of *p* < 0.001 was used, the analyses were not corrected for multiple comparisons using FWE or FDR procedures. In addition, the analyses examining the associations between Cluster C personality traits and neural responses were conducted within the SAP group (N = 18), limiting statistical power and potentially increasing the risk of Type II error and instability of the observed associations. These findings should therefore be considered exploratory and hypothesis-generating rather than definitive evidence of trait-related neural effects. Future studies with larger samples should replicate these findings using more stringent multiple-comparison correction procedures and adequately powered multivariate approaches. Fifth, the three Cluster C personality traits were examined in separate statistical models and could not be simultaneously entered into the same model. Consequently, the present analyses do not allow us to determine the unique contribution of each trait while accounting for the shared variance among Cluster C dimensions, nor do they allow us to examine potential interactive effects between personality traits. This limitation is particularly relevant given the conceptual and empirical overlap that may exist among these traits. Larger samples will be required to support multivariate approaches capable of simultaneously modelling multiple personality traits and their potential interactions. Sixth, the whole-brain analyses identify regional patterns of BOLD activation associated with the experimental conditions and personality traits but do not establish the specific functional contribution of individual brain regions. The observed regional activations should therefore be interpreted at the level of distributed neural systems rather than as evidence that a particular region performs a single specific function. Functional connectivity, network-based, or multimodal neuroimaging approaches could provide complementary information about the functional organization and interactions within these systems. Seventh, the observed activation differences cannot be interpreted unambiguously as increased neural responses in the phobic group, because they could theoretically also reflect relatively reduced responses in the control group. Moreover, the experimental design did not include a neutral baseline condition specifically optimized to isolate fear-related responses. Consequently, the direction and specificity of some neural effects cannot be established definitively. Future studies incorporating optimized baseline conditions or parametric manipulations of threat intensity could help clarify the magnitude and direction of these responses. Eighth, the nature of the neutral comparison condition represents an additional limitation. The neutral stimuli consisted of videos of wooden balls, which provided a visually simple, non-threatening baseline but did not match the phobic stimuli in biological animacy. Therefore, the phobic-versus-neutral contrast cannot fully disentangle fear-related processing from more general neural responses to biologically salient or animate stimuli. In particular, the increased recruitment of visual regions such as the fusiform gyrus and middle occipital cortex should therefore be interpreted cautiously, as these responses may partly reflect enhanced perceptual processing of complex, biologically relevant stimuli. Future studies should include non-threatening animate stimuli matched to the phobic stimuli in relevant perceptual characteristics to more specifically isolate fear-related neural responses. Finally, the cross-sectional design limits the causal interpretation of the observed associations between Cluster C personality characteristics and neural responses to phobic stimuli. Thus, the present findings do not allow conclusions about the temporal or causal direction of these relationships, including whether Cluster C personality characteristics contribute to the development or maintenance of phobic symptoms, whether persistent phobic responses are associated with the development or maintenance of personality-related characteristics, or whether both may be influenced by other factors. Longitudinal studies could examine whether personality-related neural patterns predict the persistence of phobic responses, fear extinction, or response to exposure-based interventions. Such research may also help determine whether neurobiological markers can contribute to the identification of individuals at greater risk of persistent symptoms or differential treatment response. Recent evidence suggests that neural activation patterns may also contribute to explaining differential responses to psychological interventions across culturally diverse populations, highlighting the potential clinical relevance of identifying neurobiological markers of treatment outcomes (Gkintoni & Nikolaou, 2025). A better understanding of these mechanisms could ultimately contribute to more personalized approaches to psychological intervention.

## 5. Conclusions

In summary, the present study demonstrates that neural responses to phobic stimuli in individuals with small-animal phobia are not solely determined by the characteristics of the feared stimulus but are also shaped by stable personality traits. Our findings show that Cluster C personality traits modulate the neural architecture of threat processing, related to distinct components of the fear response. In particular, avoidant traits appear to amplify perceptual vigilance and threat detection mechanisms, dependent traits are associated with alterations in cognitive control and self-referential processing, and obsessive–compulsive traits may primarily affect regulatory systems involved in behavioral control and the management of intrusive threat-related representations. These results suggest that phobic responses emerge from the interaction between stimulus-driven threat processing and trait-related personality dispositions that bias how fear-relevant information is perceived, evaluated, and regulated. From a neuroscientific perspective, this supports a view of phobia as a dynamic process involving large-scale networks integrating perceptual, emotional, and regulatory mechanisms rather than a fixed dysfunction within a single fear circuit. Beyond advancing the understanding of the neurobiological mechanisms underlying specific phobias, these findings may also have important clinical implications. Identifying how personality traits shape neural responses to threat may help inform more personalized therapeutic approaches, particularly in exposure-based treatments, by targeting individual traits that influence fear processing and emotional regulation. More broadly, these findings contribute to the growing field of personality neuroscience by showing how stable personality dispositions interact with neural threat-processing circuits. Integrating personality traits into neurobiological models of fear may therefore provide a more comprehensive framework for understanding variability in threat responses and may ultimately inform more targeted and individualized treatments for anxiety and personality-related disorders.

## Figures and Tables

**Table 1:** Descriptive statistics of the variables.

Variables	Group with Phobia N = 18			Group Without Phobia N = 21		
	%	Media	S.D.	%	Media	S.D.
Sex						
Male	27.80%			52.40%		
Female	72.20%			47.60%		
Age		37.61	11.67		22.38	6.11
SR		39.94	7.81		0.29	0.46
HARS		17.78	9.58		1.86	2.37
Avoidant PD		1.89	1.88		2.00	1.64
Dependent PD		1.44	1.04		1.43	1.08
Obsessive–compulsive PD		3.11	1.81		2.76	1.34

Note: SR = Situation–Response Inventory of Anxiousness; HARS = Hamilton Anxiety Rating Scale; PD: personality disorder.

**Table 2:** Whole-brain differences in neural processing of phobic stimuli between individuals with and without phobia.

Brain Structures	MNI Coordinates			K	F	*p*
	x	y	z			
Individuals with phobia > Controls without phobia						
L Putamen	−26	12	14	16	22.78	0.001
L Cerebellum 6	−14	−68	−14	38	21.67	0.001
L Cerebellum 6	−10	−68	−34		17.90	0.001
Vermis 6	−2	72	−22		13.91	0.001
R Fusiform Gyrus	42	−60	−14	8	20.67	0.001
L Middle Occipital	−34	−72	18	10	20.11	0.001
R Pallidum	14	0	−6	11	19.26	0.001
R Thalamus	10	−8	−2		13.54	0.001
R Parahippocampal	6	−20	−14	17	18.07	0.001
L Parahippocampal	−6	−20	−14		16.45	0.001
L Caudate	−10	20	18	9	16.85	0.001
L Thalamus	−6	−8	2	19	15.63	0.001
L Pallidum	−10	0	−6		15.34	
L Fusiform Gyrus	−30	−80	−18	3	15.60	0.001
L Middle Cingulate	−18	−32	42	3	14.93	0.001
R Anterior Cingulate	14	32	10	3	14.51	0.001
L Anterior Cingulate	−18	44	10	10	14.40	0.001
L Anterior Cingulate	−14	36	6		14.32	0.001
L Inferior Frontal pars Triangularis	−26	36	10		14.07	0.001
L Parahippocampal	−38	−44	−6	5	13.84	0.001
L Middle Temporal	−46	−48	−2		13.48	0.001

**Table 3:** Whole-brain differences in neural responses to phobic stimuli between individuals with and without phobia in models including Cluster C personality trait scores as continuous covariates.

Brain Structures	MNI Coordinates			K	F	*p*
	x	y	z			
*Individuals with phobia > Controls* ***Avoidant traits***						
R Lingual Gyrus	6	−68	6	71	19.07	0.001
L Lingual Gyrus	−14	−68	2			
R Calcarine	14	−76	6			
R Postcentral	46	−16	38	38	17.60	0.001
R Postcentral	42	−20	30			
L Lingual Gyrus	−26	−64	−6	5	16.04	0.001
R Superior Occipital	22	−60	38	6	15.86	0.001
L Postcentral	−26	−36	62	26	15.55	0.001
L Lingual Gyrus	−10	−40	−2	17	15.31	0.001
R Fusiform Gyrus	30	−80	−6	4	14.96	0.001
R Supplementary Motor Area	2	−20	54	32	14.57	0.001
L Postcentral	−46	−20	38	11	13.8	0.001
R Rolandic Operculum	46	−8	14	3	12.92	0.001
L Calcarine	−2	−84	−14	4	12.86	0.001
L Hippocampus	−18	−36	10	6	12.53	0.001
*Individuals with phobia > Controls* ***Dependent traits***						
L Middle Frontal 2	−30	16	42	14	20.15	0.001
R Calcarine	14	−80	2	43	18.03	0.001
Vermis 6						
R Middle Occipital						
R Cerebellum	22	−76	−18	22	17.38	0.001
L Precuneus	−6	−40	62	36	17.21	0.001
R Paracentral Lobule	2	−32	52			
L Insula	−34	−20	6	7	15.9	0.001
L Superior Medial Frontal	−10	32	54	8	14.8	0.001
L Superior Frontal 2	−18	28	54			
L Lingual Gyrus	−10	−84	−14	7	13.24	0.001
L Angular Gyrus	−42	−72	30	8	13.15	0.001
R Inferior Temporal	38	−52	6	3	12.03	0.002
L Rolandic Operculum	−42	−32	18	3	11.85	0.002
R Superior Frontal 2	22	24	54	4	11.72	0.002
L Superior Medial Frontal	−2	48	26	5	11.23	0.002
L Postcentral Gyrus	−46	−8	50	4	11.16	0.002
L Inferior Occipital Gyrus	−34	−80	−10	4	11	0.002
L Triangularis Inferior Frontal	−50	20	26	5	10.87	0.002
L Supramarginal Gyrus	−50	−32	26	11	10.51	0.003
L Supramarginal Gyrus	−62	−28	30		9.82	0.004
*Individuals with phobia > Controls* ***Obsessive–compulsive traits***						
R Pallidum	18	8	2	24	13.49	0.001
R Pallidum	22	0	6			
L Thalamus	−6	−20	−2	6	12.49	0.001
R Thalamus	10	−16	−2	4	12.27	0.001
L Rolandic Operculum	−50	0	10	3	11.95	0.002
R Cerebellum 6	26	−68	−30	4	11.48	0.002
R Cerebellum 6	30	−72	−22			
Vermis 7	6	−76	−26	4	11.34	0.002
R Inferior Operculum Frontal	42	8	10	4	11.19	0.002
L Putamen	−30	4	10	26	11.17	0.002
L Pallidum	−22	−4	2			
L Putamen	−30	0	−2			

## Data Availability

Data will be made available on request.

## Abbreviations

The following abbreviations are used in this manuscript:

AC–PC	Anterior Commissure–Posterior Commissure
AAL2	Automated Anatomical Labeling Atlas 2
APD	Avoidant Personality Disorder
BOLD	Blood-Oxygen-Level-Dependent
CEIBA	Comité de Ética de la Investigación y Bienestar Animal
CIDI	Composite International Diagnostic Interview
DICOM	Digital Imaging and Communications in Medicine
DPD	Dependent Personality Disorder
EPI	Echo-Planar Imaging
FA	Flip Angle
fMRI	Functional Magnetic Resonance Imaging
FOV	Field of View
FSPGR	Fast Spoiled Gradient Echo
FWHM	Full Width at Half Maximum
GLM	General Linear Model
HARS	Hamilton Anxiety Rating Scale
HRF	Hemodynamic Response Function
IPDE	International Personality Disorder Examination
MNI	Montreal Neurological Institute
MRI	Magnetic Resonance Imaging
NIfTI	Neuroimaging Informatics Technology Initiative
OCPD	Obsessive–Compulsive Personality Disorder
PD	Personality Disorder
SAP	Small-Animal Phobia
SD	Standard Deviation
SPM12	Statistical Parametric Mapping 12
S–R	Situation–Response Inventory of Anxiousness
TE	Echo Time
TR	Repetition Time
